# Anterolateral knee biomechanics

**DOI:** 10.1007/s00167-017-4494-x

**Published:** 2017-03-15

**Authors:** Andrew A. Amis

**Affiliations:** 1 Biomechanics Group, Mechanical Engineering Department, Imperial College London, London, SW7 2AZ UK; 2 Musculoskeletal Surgery Group, Department of Surgery and Cancer, Imperial College London School of Medicine, London, W6 8RF UK

**Keywords:** ACL, Anterolateral rotatory instability, Tibial internal rotation, Ilio-tibial band, Biomechanics

## Abstract

This article reviews the evidence for the roles of the anterolateral soft-tissue structures in rotatory stability of the knee, including their structural properties, isometry, and contributions to resisting tibial internal rotation. These data then lead to a biomechanical demonstration that the ilio-tibial band is the most important structure for the restraint of anterolateral rotatory instability.

*Level of evidence* V.

## Introduction

In the past, it was common practice to treat the instability that followed anterior cruciate ligament (ACL) injury by means of a lateral extra-articular tenodesis, and procedures such as those described by Lemaire and Combelles [30], Ellison [13] and Galway and MacIntosh [17] were used widely. However, the line of action of a lateral construct was far away from that of the ruptured ACL, and so it was not surprising that normal knee mechanics was not restored. A further aspect is that most authors describing the extra-articular procedures used a post-operative regime which included immobilisation in plaster of Paris, usually with the knee held in flexion and the tibia in external rotation—presumably to try to avoid ‘stretching-out’ of the tenodesis [23]. However, as may be imagined, such treatment could cause stiffness, and there was also a suspicion that it led to degenerative changes of the lateral compartment of the knee. Thus, these procedures fell from use when arthroscopy led to better and more accurate intra-articular ACL reconstruction.

Although intra-articular ACL reconstruction led to reliable restoration of anterior tibiofemoral joint laxity to normal (or close to normal) in most cases, it was also widely reported that there remained a small percentage of patients whose knees continued to feel unstable [4, 15, 19]. The symptoms reported were what, in the clinic, matched the observation of a trace of remaining pivot-shift instability, the so-called ‘pivot-glide’. It was realised that this pivot-glide represented a residual rotatory laxity. It is possible to measure the small transient instability during intra-operative pivot-shift testing post ACL reconstruction [6]. It has also been shown that increasing the ACL graft tension can cause over-constraint of tibial anterior translation laxity, yet still leave the rotational laxity greater than in the native knee [31]. Recognition of this shortcoming in some knees has led to many studies on intra-articular ACL reconstruction in recent years, in which there have been explorations of factors such as graft tension, femoral tunnel position, single-bundle versus double-bundle grafts, in attempts to abolish the troublesome residual rotational laxity.

There has recently been a renewed realisation that rotational laxity may result from injuries to the peripheral structures. This was prompted in part by an anatomy paper describing an anterolateral ligament (ALL), which received much publicity [10], but it has actually led to a return to consideration of the role of the anterolateral peripheral soft-tissue structures and of their repair or reconstruction; this paper examines the biomechanical role of these structures.

## Anterior–posterior and internal–external rotation laxity of the knee

It is well-known that the ACL is the primary restraint of tibial anterior translation [8], where ‘primary restraint’ means that, for a given anteriorly directed force applied to the proximal tibia, most of the restraint (>50%) is from the ACL. It follows that rupture of the ACL allows a large increase of tibial anterior translation laxity. That increase of laxity is greatest around 20°–40° knee flexion, and so ACL injury is best diagnosed near knee extension.

The role of the ACL in resisting tibial internal–external rotation has been less clear, and there have been papers which differ as to whether an isolated ACL rupture leads to any significant increase in tibial rotational laxity: some did find a small and significant increase in rotational laxity [33, 59], while others did not [11, 28]. This controversy probably follows from the ACL being sited centrally over the tibial plateau, and so it cannot have any large moment arm around the axis of tibial internal–external rotation, about which to resist any torque when the knee and ACL are intact or reconstructed. The axis of tibial rotation is close to the centre of the tibial plateau, usually around the medial spinous process [24]. It is usually the case, however, that an injury mechanism which ruptures the ACL includes at least some rotational component of loading and, hence, of bone–bone excursion. That is shown clearly by the presence of bone bruises on MRI, when it is common to find a bone bruise near to the centre of the distal aspect of the lateral femoral condyle, which matches the point where it impacted against the posterior rim of the lateral tibial plateau during the injury [54]. This shows clearly that the lateral aspect of the tibia had moved anteriorly from under the femur during the injury. That is not usually found to a similar degree in the medial compartment of the knee [32], and so it demonstrates that the injury had included a substantial component of tibial internal rotation.

The internal rotation which accompanies ACL injury mechanisms is a part of the normal behaviour of the intact knee. Imposing a force which translates the proximal tibia anteriorly also induces a coupled internal rotation. (A coupled motion is one which occurs automatically in response to inducing a motion in a different degree of freedom of the joint.) Data in the literature suggest that the coupled tibial internal rotation is in the range of 3°–10° when examined by hand [2, 39]; this motion may be larger under functional loading. It is usually accepted that the internal rotation which accompanies anterior translation results from the greater mobility and lack of constraint of the lateral compartment, due to both differences of meniscocapsular attachments—the lateral meniscus being more mobile than the medial—and of articular geometry—the medial tibial plateau being concave, which locates the femoral condyle, while the lateral plateau is flat or even convex in the sagittal plane.

### Effect of ACL injury in combination with anterolateral injury

If the mechanism of ACL rupture has included a large anterior translation of the lateral aspect of the tibial plateau, it follows that structures which cross the anterolateral joint line and are oriented in a direction that will resist that motion (that is: slanting from relatively posterior on the femur, across the joint to relatively anterior on the tibia) are likely also to be stretched or ruptured—a ‘combined’ injury. This has been most clearly demonstrated by the Segond fracture, when the anterolateral capsular structures cause an avulsion of a flake of bone from the rim of the tibial plateau [18]. The resulting increase in laxity of the internally rotated knee is diagnostic for anterolateral injury [53].

A combined injury of the ACL plus anterolateral structures leads to increased tibial anterior translation, and also increased tibial internal rotation laxity, when compared to the changes arising from an isolated ACL injury. This is reflected in the movements of the bones during the pivot-shift examination. Bedi et al. [5] have shown that the movement of the lateral tibial plateau is much greater than that of the medial, the difference in motion resulting from the internal rotation of the tibia as it subluxes anteriorly. A range of values for the movements of the medial and lateral condyles have been published [32]. This observation has led to a method to grade the pivot-shift which visualises the movements of the lateral aspect of the knee [36]. The overall effect is that the anterolateral aspect of the proximal tibia moves anteriorly as the internal rotation adds to the anterior translation movement during clinical examination, and that is what has long been recognised and described as ‘anterolateral rotatory instability’ (ALRI).

It should be remembered, however, that the pattern of pathological laxity is extremely variable between knees, so that some respond to the pivot-shift test with a large rotational component of motion, while others yield a predominance of anterior–posterior translation [6]. If there is a large translation of both compartments, it suggests that there has been damage also in the medial compartment, such as posteromedial menisco-capsular lesion.

Experiments in vitro, which allow the loads to be controlled accurately, have found that not only does a lesion of the anterolateral structures [which includes the capsular structures and the more proximal attachments of the ilio-tibial band (ITB)] lead to increased laxity, it also leads to a persistence of some abnormal laxity after an isolated ACL reconstruction: both anterior translation and internal rotation remain greater than when the knee was intact [21]. This observation provides a logic for using some additional anterolateral procedure to restore native knee laxity behaviour.

### Structural properties of anatomical structures at the anterolateral aspect of the knee

Recent interest has focussed on the anterolateral ligament (ALL), which has been suggested to be the structure which avulses the bone fragment in a Segond fracture [9, 18]. However, the literature has included differing descriptions of the ALL, and it has not even been found in all knees by some authors. The implication is that it is a relatively small structure, and so it is unlikely to be either strong or stiff. The result of these differences of opinion is a range of data on the structural properties of the ALL.

Zens et al. [64] found that the isolated ALL had an ultimate tensile strength of 50 ± 15 N, at a strain of 36 ± 4%. With a mean cross-sectional area of only 1.54 mm^2^, the ultimate tensile stress was 33 ± 4 MPa, and the overall stiffness was 4.2 N/mm extension. The specimens failed at mid-substance; they did not induce a bone avulsion.

Other authors have reported greater strength for the ALL, but this observation relates to the difficulty and differences in anatomical interpretation of what, exactly, is the ALL? While Zens et al. [64] separated an isolated ALL structure, it is clear that the literature has included work where the deep capsulo-osseous layer of the ITB and adjacent capsular tissue have been taken to be part of ‘the ALL’, and it may be more appropriate to view the entire anterolateral capsule-ligamentous complex as being the structure which should be reconstructed, rather than the isolated ALL. Thus, Kennedy et al. [25] reported that the ALL had a tensile strength of 175 N (139–211 N 95% CI) and stiffness 20 N/mm (16–25) with a more substantial structure than that shown by Zens et al. [64].

This strength is similar to that of the deep fibres of the medial collateral ligament (MCL), reported to be 194 ± 82 N (mean ± SD) [47], which is sheltered by the superficial MCL, the way that the ALL is protected by the ITB. The deep MCL has a significant role in restraint of tibial external rotation [48], mirroring the role of the ALL in restraining internal rotation, and may be injured by a tibial external rotation mechanism [38].

In comparison, Noyes et al. [41] reported that a graft taken from the distal part of the ITB, 18 ± 2 mm wide, had a mean tensile strength of 1068 N, and that a more proximal strip of the fascia lata 16 ± 1 mm wide had a mean tensile strength of 628 ± 35 N, reached a failure stress of 79 ± 5 MPa, and had a tensile extension stiffness of 614 ± 271 N/mm. Thus, the ITB has been reported to be stronger and stiffer than any of the measurements reported for the isolated ALL or the ALL complex which also incorporates deep capsule-osseous fibres of the ITB. Another study [45] found that a 10 mm wide strip of the ITB was 50% stronger than a 20 mm wide area of the anterolateral joint capsule.

These structural properties along the length of the ITB do not necessarily mean that it controls tibial anterolateral subluxation when the knee is nearly fully extended, because its axis is not aligned to resist that displacement, whereas it is when the knee is in flexion. However, the ITB is tethered to the lateral aspect of the femur via the Kaplan’s fibres and lateral intermuscular septum [58], and also has an anterior expansion, the lateral anterior aponeurosis, which sweeps anteriorly from the ITB, linking it to the lateral aspect of the patella, and also to the patellar tendon [35]. Thus, the ITB is set up to resist anterolateral subluxation of the tibia.

The strengths of other stabilising structures at the lateral aspect of the knee have also been reported: Sugita and Amis [57] found tensile strengths for the lateral (fibular) collateral ligament (LCL): 309 ± 91 N, and the popliteofibular ligament 186 ± 65 N. LaPrade et al. [29] found similar tensile strengths for the lateral (fibular) collateral ligament (LCL): 295 ± 96 N; popliteofibular ligament 298 ± 144 N; and popliteus tendon 700 ± 232 N.

### Length change patterns of anatomical structures at the anterolateral aspect of the knee

The literature of ACL anatomy and reconstruction has shown clearly that, because ligaments attach to bone over an area and not just at a point, there will be a spectrum of tightening–slackening behaviour across the width of the structure, due to each fibre having a different moment arm about the axis of flexion–extension. It has also been shown that the isometry depends principally on the location of the femoral graft attachment, and not the tibial [3]. These principles also apply at the lateral aspect of the knee. Although the posterior part of the lateral femoral condyle is close to being spherical, that does not mean that the axis of rotation is fixed, because knee function includes components of both rolling and sliding of the tibiofemoral joint surfaces during flexion–extension, although a trans-epicondylar axis is a good approximation.

The lateral (fibular) collateral ligament (LCL) attaches close, and just posterior, to the lateral femoral epicondyle. As the condyle rolls posteriorly, it also moves ‘downhill’ on the increasing posterior slope of the lateral tibial plateau and so the LCL slackens significantly with knee flexion [57]. Taking this as a guide, it may be imagined that a structure attaching anteriorly will be stretched as the knee flexes, and that a more-posterior attachment will cause more slackening. These length change patterns were mapped by Sidles et al. [52]. They showed that, if the area on the tibia near to Gerdy’s tubercle was taken as the datum point (which is appropriate when considering the behaviour of a lateral extra-articular tenodesis), then the matching area on the femur closest to isometric behaviour was along the posterior/lateral edge of the femoral metaphysis.

The isometry of the ALL was measured by Dodds et al. [12], by threading a suture along the ligament fibres, attaching it to the moving tibia and then measuring the changes of the separation distance between the bone attachments using a transducer. It was shown that, with the tibia following its ‘neutral’ path of motion (that is: without any control of tibial internal–external rotation while the knee was flexing/extending) the ALL was close to being isometric from 0 to 60° knee flexion, after which the length reduced, so the ALL slackened in deeper flexion. If the tibia was held in internal rotation, the ALL was longer across the arc of motion, particularly in the flexed knee, showing that it was stretched and, therefore, would be resisting tibial internal rotation. This ‘close to isometric’ behaviour was associated with the femoral attachment being identified in that work as proximal and slightly posterior to the lateral epicondyle. Although the exact measurement varied between knees, the mean position was 8 ± 5 mm SD proximal and 4 ± 5 mm posterior to the tip of the epicondyle. A similar femoral attachment position has been reported in another study [25], at a mean of 7 mm from the lateral epicondyle. In contrast to the above studies, Zens et al. [65] reported that the ALL attached antero-distal to the lateral epicondyle, and this difference of anatomical identification led to the ALL being reported to be stretched by knee flexion and slack near extension in that study, a characteristic which would not help to stabilise the knee when weight-bearing.

Noting the differing findings of isometric behaviour, isometry of anatomical structures and of several of the points published for lateral extra-articular tenodeses was measured by Kittl et al. [26], using similar methods to the work by Dodds et al. [12]. The results confirmed that the ALL was close to isometric, with slight tightening (that is lengthening of the distance between the bone attachments) as the knee was extended. This behaviour was identified as being most desirable for a lateral tenodesis or similar procedure, because it would need to be tight to resist the pivot-shift subluxation close to knee extension (the weight-bearing posture), yet slacken in knee flexion so as not to over-constrain the increased tibial internal–external rotation laxity of the native undamaged knee. Conversely, if a lateral tenodesis were to be fixed antero-distal to the lateral femoral epicondyle, it would slacken as the knee extended, and so be unable to resist the anterolateral subluxation of the pivot-shift.

An important practical finding in the work of Kittl et al. [26] was that if a lateral tenodesis graft was taken from the area of Gerdy’s tubercle and then routed deep to the LCL for attachment to the femur, as described in the Galway and MacIntosh tenodesis [17, 23], then the proximal femoral attachment of the LCL acted as a pulley and kept the graft behaviour close to isometric, as described above for the ALL, with graft elongation with knee extension, a desirable characteristic for stabilising the knee. It was found that it did not matter where along the lateral condylar ridge the graft was attached, the isometric behaviour remained very similar. This finding allows the surgeon to choose where to place the fixation along the lateral femur, possibly to avoid interacting with an ACL graft tunnel, for example.

### Role of structures to resist ALRI and tibial internal rotation

Recent work has found that, in the presence of an injury which includes damage to both the ACL and the anterolateral structures, an isolated ACL reconstruction left a residual abnormal rotational laxity of the tibiofemoral joint [21]. In response, biomechanical studies have examined the effects of cutting individual anatomical structures around the knee, to demonstrate their roles in constraint of tibial rotation. When the effect of an isolated ACL deficiency is studied, the changes in tibial internal rotation laxity may be small enough that it is difficult to find a significant change in tibial rotation in response to a simulated pivot-shift test, between intact, ACL-deficient, and ACL-reconstructed states [11]. Because the pivot-shift test usually involves application of a valgus moment to the knee, there is a tendency for it to induce a coupled tibial internal rotation, due to the compressive load in the lateral compartment and then the associated tendency of the femoral lateral condyle to slip ‘downhill’ down the posterior slope of the lateral tibial plateau [42].

Changes in rotational laxity are clearer when there is a combination of ACL plus peripheral lesions. Thus, Spencer et al. [56] showed that the mean coupled internal rotation during a simulated pivot-shift test increased from 5° when the knee was intact to 7° after isolated ACL transection, to 9° when the ALL was cut. Other work [55] reported greater coupled internal rotation during the simulated pivot-shift test, from 18° intact, to 20° ACL cut, 26° ACL + ALL cut, to 35° with ACL + ALL + ITB cut. They found greater laxity when an isolated 2 N m tibial internal rotation torque was applied: 30–32–35–42°, for the same cutting sequence, at 20° knee flexion. Another study [46] found that cutting the ACL led to increased tibial internal rotation from 0 to 45° knee flexion, but only up to 2° increases. In contrast, adding the ALL cut led to a further 3°, across 0 to 120° knee flexion. These papers showed clearly that cutting the ALL led to significant increases in tibial internal rotation laxity, which means that the ALL must act to resist tibial internal rotation. In contrast to these studies, it has also been reported that adding an ALL lesion to an ACL-deficient knee did not lead to measureable increases in either anterior translation or internal rotation during clinical testing of whole cadavers [50].

However, although changes of internal rotational laxity are what are examined and may be diagnostic for specific ligament injuries during a clinical evaluation of a knee, the resulting data do not tell us how much of the applied load (internal rotation torque in the case of testing for ALRI) has been resisted by each of the relevant anatomical structures. To do that—to discover which structures are the primary restraints—a different type of test has to be used, in which the changes of load are measured when a test that displaces the tibia a constant amount is repeated after cutting a structure.

The method of sequential cutting of anatomical structures was introduced by the work of Butler et al. [8] which led to identification of the cruciate ligaments as being the primary restraints to tibial anterior and posterior translation. They held the femur and tibia in a materials testing machine and displaced the tibia a known distance and recorded the force required. They then repeated that anterior translation after cutting the ACL, and the reduction in force was what had been resisted by the ACL. That simple method, however, held the bones rigidly and prevented secondary movements. In particular, release of tibial internal rotation was found to allow 30% more anterior translation [16]. So test rigs with many sliding and rotating bearings were developed, to allow multiple degrees of freedom of motion when the tibia was displaced, and led to detailed data on the roles of the ligaments (for example, for the PCL bundles [44]). The problem then was that the repetition of the drawer force could not ensure that the tibia followed the same path of motion (the cut structure might have altered the internal rotation during a draw test, for example), and so the forces in the remaining ligaments would change, affecting the data on their contributions. This dilemma was solved by the introduction of robotic tests of knees, because the robot would record the path of motion during a test of the intact knee, and could reproduce it precisely after a ligament had been cut [49].

There have been several in vitro studies of internal rotation of the knee which used robotic technology. One study [46] used the robot in order to measure the increases of laxity when the ACL and then ALL were cut, across the arc of knee flexion, but did not report using the load sensor to also discover how those cuts affected the loads on the knee during the movements. Another in-vitro study [43] found that the ACL resisted 35% of a 5 N m internal rotation torque near knee extension, falling to 20% at 60° flexion, with the converse trend for the ALL: rising from 5% near extension to 40% at 60° knee flexion. Unfortunately, this experiment was performed after the ITB had been removed, and so the percentage contributions would have been over-stated, because the ITB has since been shown to be the primary restraint (see below). Thein et al. [61] found that the ALL had resisted <10 N force during a tibial anterior translation test, and <17 N during the simulated pivot-shift test; thus, they concluded that the ALL could only be considered a secondary restraint.

**Fig. 1:**
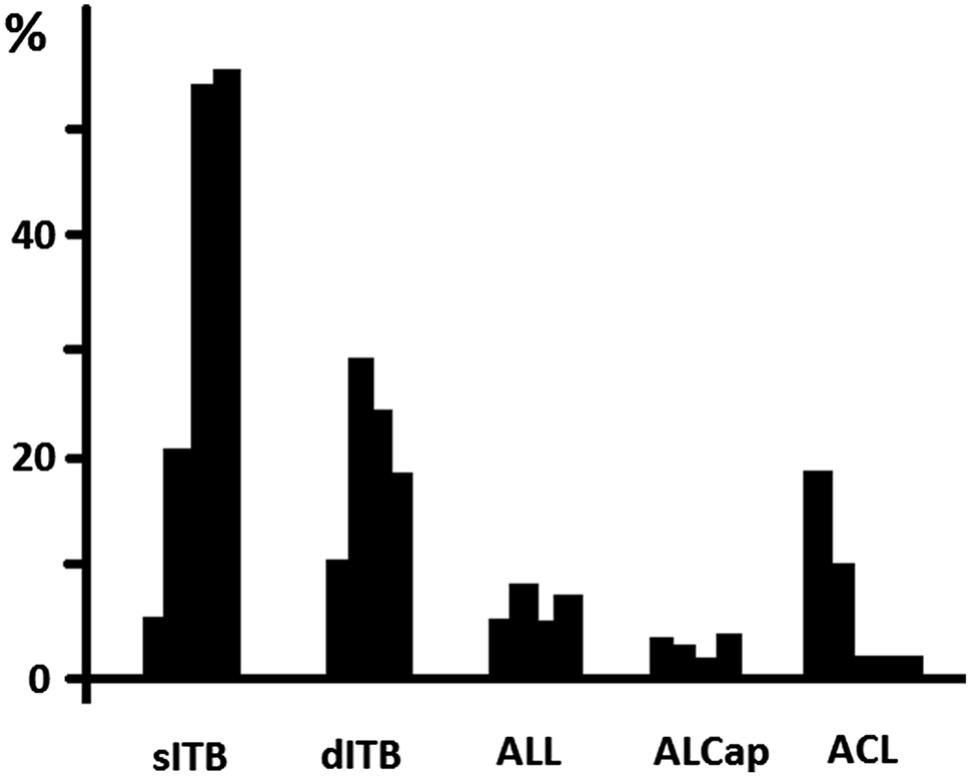
Mean contribution (%) of tested structures in restraining a 5 N m internal rotation torque at 0°, 30°, 60°, and 90°. *sITB* superficial layer of the iliotibial band, *dITB* deep and capsulo-osseous layer of the iliotibial band, *ALL* anterolateral ligament, *ALCap* anterolateral capsule, *ACL* anterior cruciate ligament. (Based on data from Kittl et al. [26])

The study by Kittl et al. [27] measured the reductions in tibial internal rotation torque during a sequence of cuts of the anterolateral structures: the superficial ITB, the deep/capsule-osseous fibres of the ITB; the ALL, then the anterolateral joint capsule. This was done for knees with the ACL intact, and also for ACL-deficient knees. The main finding of this study was that it was the ITB which was the primary restraint to tibial internal rotation (Fig. 1). Also, contrary to the recent opinion based on anatomic observations and laxity changes, neither the extracapsular ALL nor the anterolateral capsular structures offered significant resistance to tibial internal rotation from 0 to 90° knee flexion. At full knee extension, where it becomes tight, the ACL was a significant restraint in the intact knee, but that contribution fell rapidly with knee flexion, as the more-posterior fibres of the ACL slackened. In an ACL-deficient knee, the resistance which had been taken by the ACL was then taken by an increased contribution from the deep capsulo-osseous layer of the ITB. When the contributions of the superficial and deep layers of the ITB were added together, the ITB was found to have been the primary restraint (that is: it resisted more than 50% of the torque) to tibial internal rotation above 30° knee flexion, and this contribution increased with knee flexion, to 74% of the total resistance at 60° knee flexion.

**Fig. 2:**
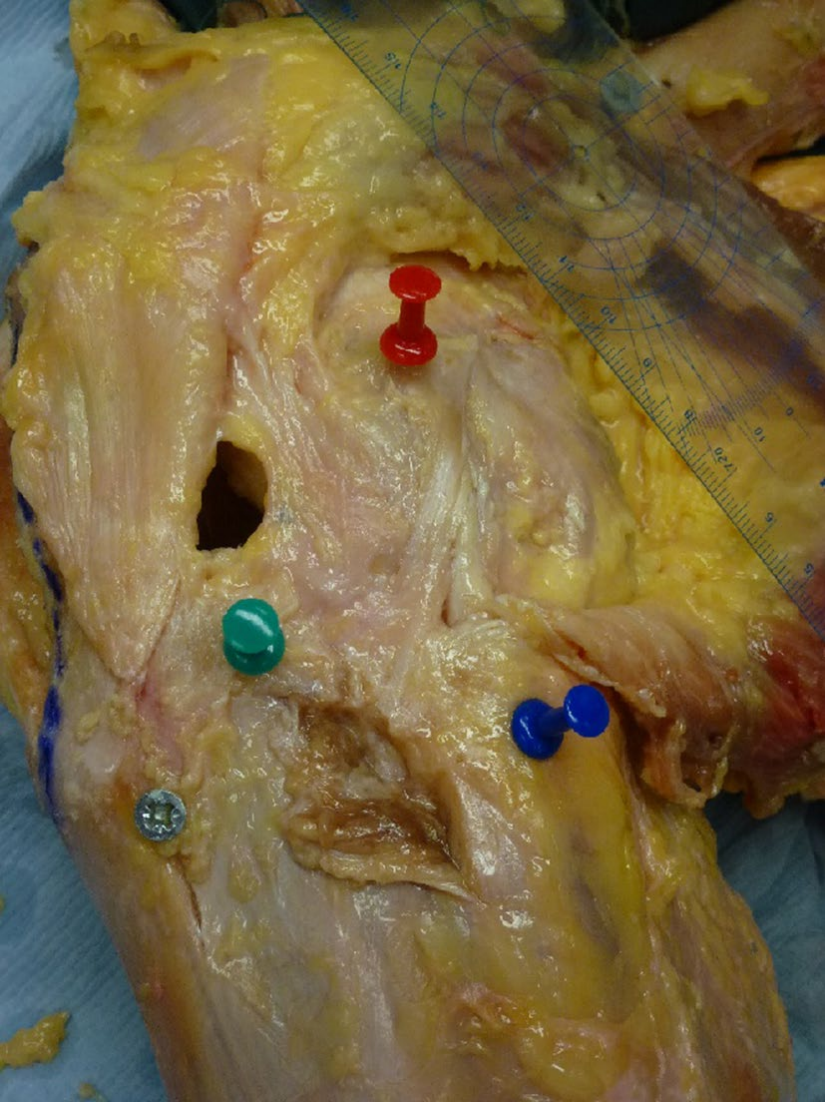
Dissection to show the anterolateral ligament (ALL) as described by Dodds et al. [12]. View of the anterolateral aspect of a left knee at 90° flexion. The *red pin* is at the lateral femoral epicondyle; the *blue pin* is at the distal attachment of the lateral (fibular) collateral ligament (LCL); the *green pin* is at Gerde’s tubercle. The ALL passes superficially over the proximal part of the LCL and attaches mid-way between Gerde’s tubercle and the head of the fibula

The data from Kittl et al. [27] fits with the operative observations of Terry et al. [59, 60], who found injury of the deep capsulo-osseous layer of the ITB in 93% of the functionally unstable knees that they reconstructed. They also reported that this ITB damage correlated significantly with the grade of the pivot-shift, whereas ACL damage did not. Similarly, it has been reported [37] that the pivot-shift laxity was increased in knees with anterolateral soft-tissue injury identified on MRI, in relation to knees with isolated ACL rupture. Work related to double-bundle ACL reconstruction has found that the posterolateral bundle of the ACL has a role in controlling tibial internal rotation at low flexion angles [63], and this fibre bundle is known to slacken with knee flexion [1]. It has long been known that the ITB shares the resistance to anterior draw of the internally-rotated tibia approximately equally with the ACL [40]. It may be concluded that the role of the ITB as a restraint to ALRI might have been underestimated in the recent literature, which has focussed mostly on intra-articular ACL reconstruction, and then the more recent attention on the ALL, which is a comparatively flimsy and compliant structure (Fig. 2). Yamamoto et al. [62] and Hassler and Jakob [20] found that cutting the ITB caused a large increase in internal rotation, and that the reduction of the lateral tibial plateau in a pivot shift test disappeared. That was presumably caused by the loss of the posteriorly directed component of the tension in the ITB, which increases as knee flexion increases, until it is able to suddenly reduce the tibia beneath the lateral condyle of the femur [7, 34].

### Implications for surgical treatment

The biomechanical evidence reported above suggests that a combined intra-plus extra-articular procedure may be appropriate when damage to both the ACL and peripheral structures has been identified. Recent work in vitro [21] has found that, in the presence of a combined injury, an isolated ACL reconstruction allowed a residual abnormal laxity in both anterior translation and internal rotation, and that the addition of a lateral extra-articular tenodesis abolished that deficit. Engebretsen et al. [14] found that an extra-articular graft shared the load on an ACL reconstruction, so may help to protect it during graft remodelling.

**Fig. 3:**
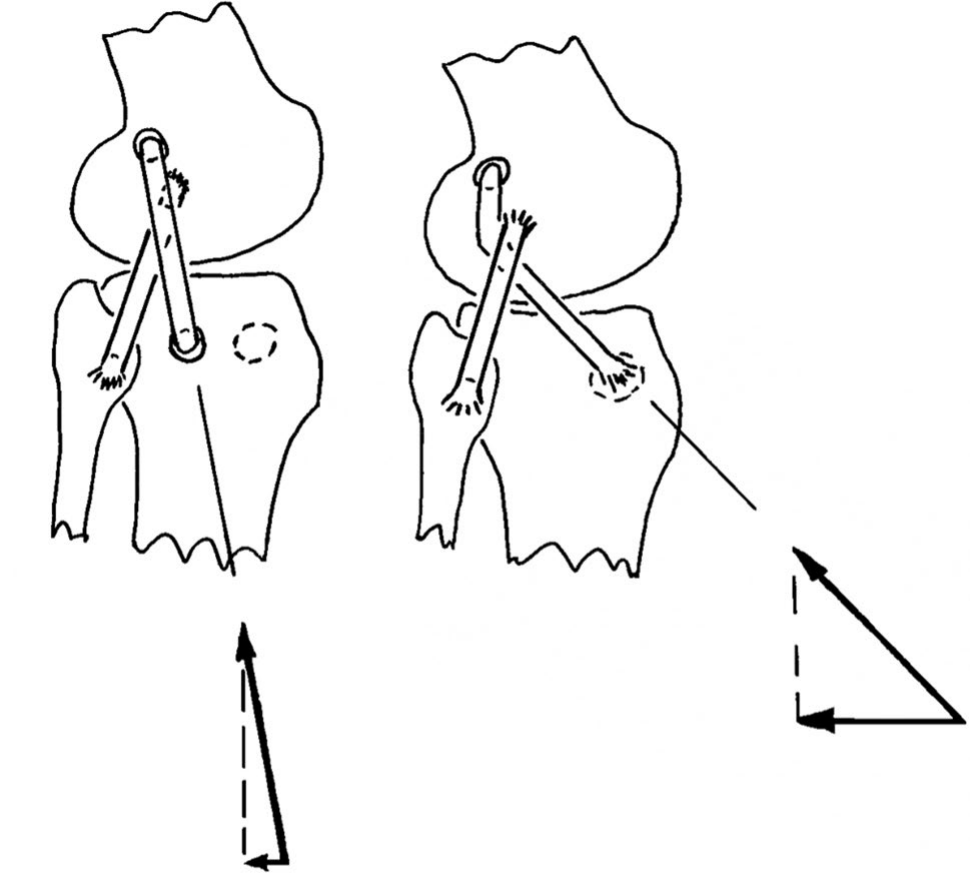
*Left* A graft placed at the attachments of the anterolateral ligament (ALL) has a relatively steep orientation as it crosses the tibio-femoral joint line, thus a graft tension produces a small posterior component of force to resist tibial internal rotation. *Right* The more-anterior attachment of a tenodesis based on Gerdy’s tubercle creates a more efficient orientation to restrain tibial internal rotation than a procedure based at the tibial attachment of the ALL, particularly if the graft is passed deep to the LCL, which then acts like a pulley. In this diagram, the posterior force vector is four times larger in the *right* diagram than in the *left*, yet the graft tensions are the same

**Fig. 4:**
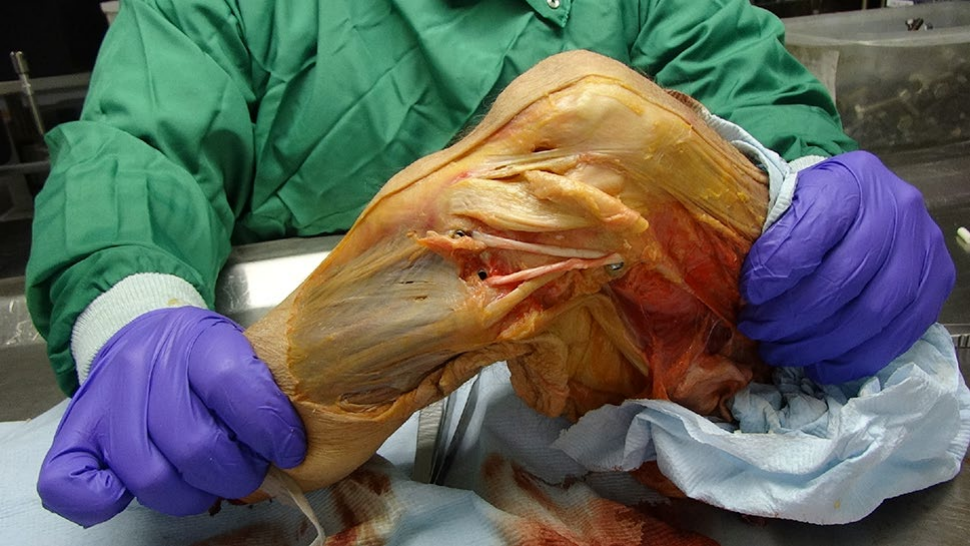
This picture of the anterolateral aspect of a left knee held with an internal rotation torque applied to the tibia shows clearly that the more-anterior graft attached at Gerde’s tubercle is better-oriented to resist tibial internal rotation than is the more-posterior arm of the graft, which simulates the line of action of the anterolateral ligament

If an anterolateral procedure is considered in combination with an ACL reconstruction, it is worth noting that the biomechanical evidence derived from the natural soft tissue structures points towards a tenodesis or reconstructive procedure that aims to recreate the restraint provided by the ITB, rather than that of the ALL. In addition to the data on percent restraint to tibial internal rotation shown in Fig. 1 [27], the data on tensile stiffness show that a tibial displacement which stretches the anterolateral structures will cause the restraining tension to rise more rapidly in the ITB. Based on the data reported above, a 5-mm elongation would lead to an ITB tension of 3070 N (stiffness 614 N/mm reported by Noyes et al. [41]), while the ALL tension would reach 100 N (stiffness 20 N/mm reported by Kennedy et al. [25]). Furthermore, this disparity in tensions is compounded by the directions in which the tensions act, as they cross the joint line: Gerdy’s tubercle is approximately 18 mm anterior to the tibial attachment of the ALL [12], and so a tenodesis based there will have a larger anterior–posterior component of the tension acting to resist the tibial internal rotation than would the tension in an ALL reconstruction (Fig. 3). Thus, although the ALL has a role in restraining tibial internal rotation, the biomechanical data suggest that an ITB tenodesis based on Gerdy’s tubercle will be more efficient (Fig. 4).

There has recently been a report that a lateral extra-articular procedure led to over-constraint of tibial internal rotation laxity across the arc of knee flexion [51]. However, that study used a graft tension of 88 N. A study of anterolateral tenodeses in the author’s laboratory [22] found that a graft tension of 20 N with the foot held in neutral rotation and the knee at 30° flexion restored native knee laxity, while significant over-constraint and elevated lateral compartment contact pressures resulted from 80 N graft tension, particularly if the tibia was left free to move into external rotation when the graft was fixed.

However, judgement as to which cases may be suitable for a combined ACL plus lateral extra-articular procedure is beyond the scope of this paper on the biomechanics of the natural structures at the anterolateral aspect of the knee; and then which of the possible lateral procedures will give the best clinical results, also remains a matter for debate, which requires collection of more outcomes data.

## Conclusions on anterolateral knee biomechanics

The biomechanical evidence reported above leads to some observations which may guide the surgeon:

- Although the ACL is the primary restraint to anterior translation of the tibia, its central location means that it has a very small moment arm to control tibial internal rotation.
- Tibial internal rotation is resisted primarily by the ITB, which is the primary restraint as the knee flexes. The lateral extra-articular structures have the largest moment arm to resist tibial internal rotation.
- Internal rotation laxity increases with knee flexion in the native knee, and is increased further by soft-tissue injuries. Isolated rupture of the ACL causes a very small increase of internal rotation, so an obvious increase of internal rotation laxity implies damage to other structures.
